# Characterization and specificity of the linear epitope of the enterovirus 71 VP2 protein

**DOI:** 10.1186/1743-422X-9-55

**Published:** 2012-02-24

**Authors:** Tanja K Kiener, Qiang Jia, Xiao Fang Lim, Fang He, Tao Meng, Vincent Tak Kwong Chow, Jimmy Kwang

**Affiliations:** 1 Animal Health Biotechnology, Temasek Life Sciences Laboratory, National University of Singapore, 117604 Singapore, Singapore; 2 Department of Microbiology, Yong Loo Lin School of Medicine, National University of Singapore, 117597 Singapore, Singapore

**Keywords:** Hand, foot and mouth disease, Enterovirus 71, Coxsackievirus A16, VP2 capsid protein, Linear epitope, Monoclonal antibody, Antigen capture ELISA

## Abstract

**Background:**

Enterovirus 71 (EV71) has emerged as a major causative agent of hand, foot and mouth disease in the Asia-Pacific region over the last decade. Hand, foot and mouth disease can be caused by different etiological agents from the enterovirus family, mainly EV71 and coxsackieviruses, which are genetically closely related. Nevertheless, infection with EV71 may occasionally lead to high fever, neurologic complications and the emergence of a rapidly fatal syndrome of pulmonary edema associated with brainstem encephalitis. The rapid progression and high mortality of severe EV71 infection has highlighted the need for EV71-specific diagnostic and therapeutic tools. Monoclonal antibodies are urgently needed to specifically detect EV71 antigens from patient specimens early in the infection process. Furthermore, the elucidation of viral epitopes will contribute to the development of targeted therapeutics and vaccines.

**Results:**

We have identified the monoclonal antibody 7C7 from a screen of hybridoma cells derived from mice immunized with the EV71-B5 strain. The linear epitope of 7C7 was mapped to amino acids 142-146 (EDSHP) of the VP2 capsid protein and was characterized in detail. Mutational analysis of the epitope showed that the aspartic acid to asparagine mutation of the EV71 subgenogroup A (BrCr strain) did not interfere with antibody recognition. In contrast, the serine to threonine mutation at position 144 of VP2, present in recently emerged EV71-C4 China strains, abolished antigenicity. Mice injected with this virus strain did not produce any antibodies against the VP2 protein. Immunofluorescence and Western blotting confirmed that 7C7 specifically recognized EV71 subgenogroups and did not cross-react to Coxsackieviruses 4, 6, 10, and 16. 7C7 was successfully used as a detection antibody in an antigen-capture ELISA assay.

**Conclusions:**

Detailed mapping showed that the VP2 protein of Enterovirus 71 contains a single, linear, non-neutralizing epitope, spanning amino acids 142-146 which are located in the VP2 protein's E-F loop. The S/T(144) mutation in this epitope confers a loss of VP2 antigenicity to some newly emerged EV71-C4 strains from China. The corresponding monoclonal antibody 7C7 was used successfully in an AC-ELISA and did not cross-react to coxsackieviruses 4, 6, 10, and 16 in immunofluorescence assay and Western blots. 7C7 is the first monoclonal antibody described, that can differentiate Coxsackievirus 16 from Enterovirus 71.

## Background

Human enterovirus 71 is a member of the enterovirus A species within the genus *Enterovirus *of the family *Picornavirus. Picornaviridae *are small (30 nm), non-enveloped, single-stranded RNA viruses that are responsible for a variety of communicable diseases in humans such as poliomyelitis, hepatitis A, the common cold as well as hand, foot and mouth disease (HFMD). Enteroviruses are distinguished from other picornaviruses on the basis of their physical properties and encompass polioviruses, rhinoviruses, echoviruses, coxsackieviruses and the "EV" enteroviruses. The human enteroviruses are now classified into 4 species: human enterovirus A (HEV-A) including coxsackievirus CAV4, 6, 10 and 16 and EV71, HEV-B, HEV-C, and HEV-D [1]. Since its first description in 1974 [2], there were periodic outbreaks of EV71 infection throughout the world. Over the last decade, EV71 has become endemic in the densely populated Asia-Pacific region, and epidemic outbreaks of HFMD occur frequently in Singapore, Taiwan, Malaysia, and China, raising concerns that the virulence and prevalence of EV71 may be increasing [3]. Furthermore, rapid mutation rates result in the emergence of new subgenotypes every few years [4]. To date, 11 EV71 subgenogroups have been identified based on comparison of their VP1 sequence: A, B1-B5, C1-C5 [5]. The Asian pandemics have been associated with co-circulation of different genetic lineages and the emergence of novel strains [6-9].

Although EV71 infection mainly manifests as HFMD in young children, the potential of enteroviruses to attack the central nervous system was first witnessed in a large epidemic in Bulgaria (1975) and Hungary (1978) where prominent neurologic manifestations such as aseptic meningitis, brainstem encephalitis and acute flaccid paralysis were observed [10,11]. HFMD can be caused by different etiological agents of the enterovirus family, mainly EV71 and CA16 [12] and molecular studies have shown a close genetic similarity between these two viruses [13]. Nevertheless, infection with EV71 more often leads to high fever and neurologic complications in children under 5 years of age [14-16] and the case-fatality rate of EV71 infection with complications ranges from 10% to 26% [17]. Especially worrying was the emergence of a rapidly fatal syndrome of pulmonary edema associated with brainstem encephalitis [18]. The occurrence of more frequent EV71 pandemics associated with severe neurological disease and fatalities has highlighted the need for EV71-specific diagnostic and therapeutic tools.

The EV71 virus particle consists of a naked icosahedral capsid composed of the four structural proteins VP1-4 surrounding a single-stranded positive-strand RNA of about 7.4 kb. The viral RNA contains a single open reading frame coding for a polyprotein which is autocatalytically cleaved after translation. P1, P2, and P3 are three distinct regions on the polyprotein that encode the structural proteins (P1) and the seven accessory proteins 2A-C and 3A-D (P2 & P3). The functions of these 11 EV71 proteins are thought to be identical to those described for poliovirus and other enteroviruses [19]. The polyprotein precursor is processed during translation and the primary cleavage of P1 and P2 is mediated by 2A^pro^. Next, 3CD^pro ^is released from P3 by autocatalysis and proceeds to cleave P1 into the capsid proteins VP0, VP1, and VP3. These three structural proteins self-assemble stepwise into empty capsids. Once the genomic RNA is loaded into the capsid, the provirion matures into an infectious virion through the cleavage of VP0 into VP2 and VP4. As the VP0 scissile bond is located inside the provirion, it is inaccessible to viral or cellular proteases. The cleavage is thought to be mediated by RNA but the exact process is yet unidentified. However, recent data confirms the presence of 3CD^pro ^in the virus particle (in press), making it a novel candidate for this cleavage. The three major capsid proteins VP1, VP2, and VP3 have the same topology: They form an eight-stranded anti-parallel beta-barrel in the form of a wedge which facilitates packing. Their main structural differences are the connecting loops and the C-termini on the outside of the capsid. VP4, however, has an extended conformation and is found on the inside of the virion. It is myristylated and confers stability to the capsid [20]. The VP1 capsid protein harbors the main neutralizing epitopes of EV71 [21-24] and its sequence variability is used to classify EV71 into subgenogroups [25]. In addition, neutralizing or antigenic epitopes on the VP0 and VP2 proteins have been described in other members of the picornavirus family including poliovirus [26,27], coxsackievirus A9 [28], foot-mouth-disease virus [29], and parechovirus [30]. Furthermore, VP0 has been proposed as a diagnostic tool to detect anti-human parechovirus 1 antibodies in patient sera [31,32].

Due to the increasing number and severity of EV71 infections, diagnostic monoclonal antibodies (MAbs) to directly detect EV71 virions from patient swabs are urgently needed. To create such MAbs we have screened hybridoma cells derived from mice immunized with the EV71-B5 strain and isolated the MAb designed 7C7. EV71-B5 (NCBI accession # FJ172159.1) has been isolated from a patient in the large 2008 outbreak of HFMD in Singapore. The epidemic was characterized by high transmission but relatively mild disease, coinciding with the transition of the EV71 subgenogroup from B4 to B5 [33]. Mutational analysis, Western blotting and immunofluorescence assay (IFA) were used to characterize the epitope of 7C7 and its specificity in detail. We successfully employed 7C7 to detect different EV71 subgenotypes and demonstrated the absence of cross-reactions to CAV16 in both IFA and Western blots. Additionally, we performed an antigen-capture ELISA (AC-ELISA) using polyclonal anti EV71 serum as capture antibody and 7C7 as detection antibody.

## Results

### Monoclonal antibody production against EV71 VP2 protein

**Figure 1:**
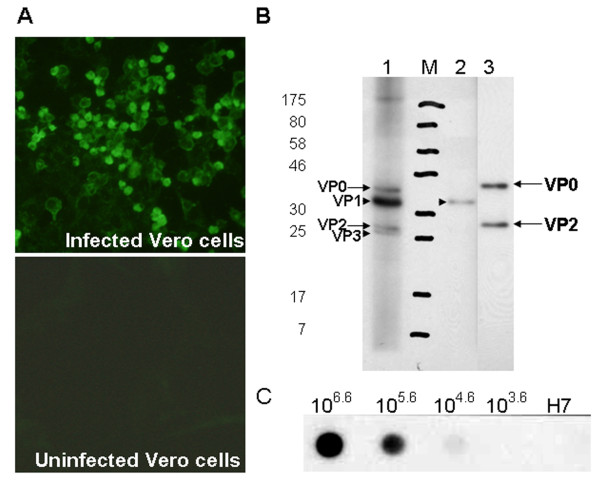
**Characterization of MAb 7C7**. IFA (A), Western blot (B), Dot blot (C) of EV71 virus with MAb 7C7. (A) IFA of EV71 infected Vero cells. Cells were labeled with MAb 7C7 followed by anti-mouse FITC secondary antibody. Top panel: Vero cells infected with EV71-B5 (NUH0083). Bottom panel: Non-infected Vero cells. (B) Western blot of sucrose purified EV71-C4 strain (75-Yamagata-03). Blots were labeled with primary antibodies and HRP coupled secondary antibodies and bands were developed by ECL. Lane 1: Polyclonal guinea pig anti-EV71 (B5 strain) antibody detects all three capsid proteins VP0/2 (arrows), VP1 and VP3 (arrowheads). Lane 2: Monoclonal anti-VP1 antibody (in press) specifically labels the VP1 band (arrowhead). Lane 3: MAb 7C7 recognizes VP2 at about 28 kDa and its precursor VP0 at around 35 kDa (arrows). (C) Sensitivity of MAb 7C7 in a dot blot assay. EV71-B5 virus was dotted at 10-fold dilutions of TCID_50 _units per dot, labeled by MAb 7C7 and detected by HRP-coupled secondary antibody. H7N1 virus served as negative control. The detection limit was around 10^5 TCID_50 _units.

Monoclonal antibodies against inactivated virus were developed. Mice were immunized subcutaneously with 100 μl of concentrated EV71-B5 strain virus in a 1:1 emulsion with Seppic adjuvans. Two boosters were administered at 14 day intervals, followed by a final intraperitoneal boost. Splenocytes were then harvested and fused with hybridoma cells according to standard protocols. Monoclonal antibody screening by IFA and subsequent subcloning resulted in the isolation of the VP2-specific MAb 7C7. The MAb was isotyped as IgG1 with kappa light chain by Isostrip (Santa Cruz). The antibody was able to detect EV71 in infected African green monkey kidney cells (Vero) as observed by IFA. No labeling was observed in uninfected cells (Figure 1A). Western blotting of EV71 virus particles from the C4 (Yamagata) strain revealed that the MAb 7C7 was specific for VP2 at 28 kDa, and also recognized the precursor protein VP0 at around 35 kDa (Figure 1B). Polyclonal guinea pig anti-EV71 serum and monoclonal anti-VP1 antibody (in house production) were used as positive controls. To test the sensitivity of MAb 7C7, a dot blot assay was performed. EV71-B5 virus was dotted at dilutions of 10^6.6 ^to 10^3.6 ^TCID_50 _units per dot, and labeled with 7C7. Purified influenza virus H7N1 served as a negative control. The detection limit was around 10^5 ^TCID_50 _units (Figure 1C). No neutralization activity was observed in a virus neutralization assay.

### Epitope mapping of 7C7 anti-VP2 MAb

**Figure 2:**
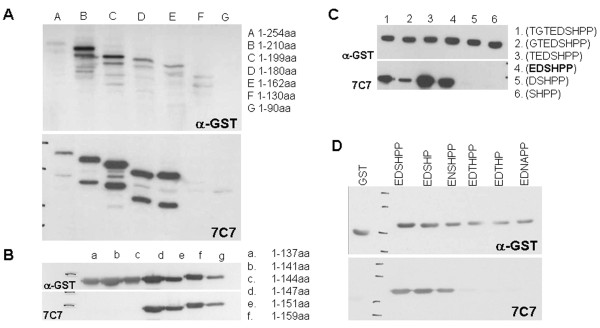
**Epitope Mapping of MAb 7C7**. (A) Fragmentation of the VP2 protein into 7 continual overlapping segments. Western blotting of the fragments with anti-GST showed expression of all constructs, while blotting with 7C7 did not recognize fragments F and G, indicating that the epitope lies between amino acids 130 and 162 of VP2. (B) Fragmentation of amino acids 1-159 of VP2 into 6 continual overlapping segments. Again, blotting with anti-GST confirms protein expression, while blotting with 7C7 indicates that the epitope lies on fragment "d" between amino acids 145-159 of VP2. (C) Fragmentation of "d" into 6 continual overlapping peptides. Anti-GST blotting shows expression while the epitope of 7C7 is restricted to peptide "4" corresponding to amino acids EDSHPP. (D) Mutational analysis of the putative 7C7 epitope EDSHPP: Mutated epitopes were expressed as GST fusion proteins and expression was confirmed by Western blotting with anti-GST antibody. The minimal epitope EDSHP as well as the aspartic acid to asparagine substitution (E**N**SHPP) present in EV71 BrCr strain and CAV16 could be recognized by our MAb 7C7. However, the threonine to serine mutation (ED**T**HPP) of some C4 strains and the double mutation of human echoviruses (ED**NA**PP) could not be recognized.

To characterize the 7C7 epitope, the VP2 protein was fragmented into GST-tagged continual overlapping peptides, cloned into pGex-4 T-1 vector and expressed in *E. coli *BL21 cells. Expression of the fusion proteins was monitored by Western blotting with anti-GST. By gradual reduction of the fragment length, the epitope location was narrowed down from amino acids 130 to 162 (Figure 2A) to fragment "d" between amino acids 145-159 (Figure 2B) and finally to peptide "4" corresponding to amino acids EDSHPP (Figure 2C). This sequence was subjected to BLAST analysis against complete EV71 sequences in the NCBI database, and two single amino acid variations were identified. An aspartic acid to asparagine substitution (E**N**SHPP) was present in the EV71-A (BrCr strain) subgenogroup and CAV16 while a threonine instead of serine (ED**T**HPP) was present in some EV71-C4 China strains. Furthermore, the human echovirus epitope contained a double substitution of serine to asparagine and histidine to alanin (ED**NA**PP) at positions two and three, respectively (Table 1). We expressed these putative epitopes EDSHPP, EDSHP, ENSHPP, EDTHPP, EDTHP, and EDNAPP as GST-fusion proteins, and assessed recognition by MAb 7C7. Expression of GST fusion peptides showed that the minimal epitope of 7C7 were the five amino acids EDSHP which correspond to positions 142-146 of the EV71 VP2 capsid protein (Figure 2D, lane 4). 7C7 was also able to detect the EV71-A (BrCr) and CAV16 epitope variant ENSHPP (Figure 2D, lane 5). There was no recognition of the echovirus double mutant EDNAPP, confirming the exclusive specificity of 7C7 to EV71 and maybe CA16 (Figure 2D, lane 8). To our surprise, the EDTHP epitope variant, present in some EV71-C4 China strains, was not recognized by 7C7 (Figure 2D, lane 7). By constructing a phylogenetic tree of published EV71 sequences, we could demonstrate that the 30 EV71 sequences containing the serine to threonine substitution all belong to the C4 subgenogroup isolated in recent years (2008 - 2010) in China (Table 2). This includes the C4-Fuyang-08 strain which was engineered in our lab by reverse genetics [34].

**Table 1:** Alignment of VP2 amino acid (131-155) sequences of EV71 subgenogroups and CAV16

Genogroup	Strain	GenBank #	Sequence
C4	Fuyang-08	EU703813.1	VIGTVAGGTGTED**T**HPPYKQTQPGA

C4	75-Yamagata	AB550338.1	VIGTVAGGTGTEDSHPPYKQTQPGA

C2	NUH0075/SIN/08	FJ172159.1	VIGTVAGGTGTEDSHPPYKQTQPGA

B2	7423-MS-87	U22522.1	VIGTVAGGTGTEDSHPPYKQTQPG

B4	5865/SIN/09	AF316321.2	VIGTVAGGTGTEDSHPPYKQTQPGA

B5	NUH0083/SIN/08	FJ461781.1	VIGTVAGGTGTEDSHPPYKQTQPGA

A	BrCr	U22521.1	VIGTVAGGTGTE**N**SHPPYKQTQPGA

CAV16	CAV16	U05876	VLGTIAGG**D**G**N**E**N**SHPPY**VT**TQPGQ

**Table 2:** EV71-C4 strains with S/T(144) mutation

Year	GenBank #	Year	GenBank #	Year	GenBank #
2010	ADX87405.1	2010	ADX36154.1	2010	ADR73044.1

2010	ADG57604.1	2010	ADJ37050.1	2010	ADJ37049.1

2010	ADI49646.1	2010	ADI49645.1	2010	ADI49642.1

2010	ADC84177.1	2010	ADC84176.	2010	ADC54995.1

2010	ACY00665.1	2010	ACY00664.1	2009	ACX46121.1

2009	ACU45381.1	2009	ACU45378.1	2009	ACU45377.1

2009	ACM62758.1	2009	ACM62757.1	2009	ACJ70062.1

2008	ACJ04794.1	2008	ACJ04792.1	2008	ACI25379.1

2008	ACI03062.1	2008	ACI03061.1	2008	ACF60581.1

2008	ACF21980.1	2008	ACD63040.1	2008	ACD63039.1

2008	ACD63041.1				

### Specificity of 7C7 to EV71 subgenogroups

**Figure 3:**
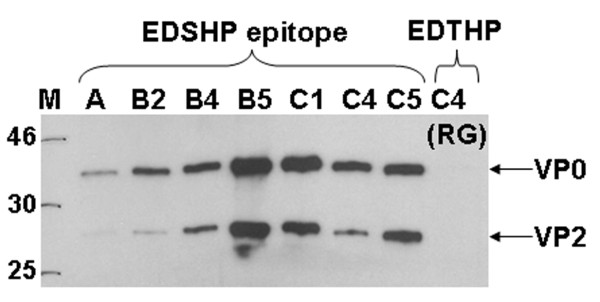
**Specificity of 7C7 to EV71 subgenogroups**. Western blot of concentrated virus particles from EV71 strains A (BrCr), B2 (7423-MS-87), B4 (HFM41), B5 (NUH0083-SIN-08), C1 (Y90-3761), C4 (75-Yamagata-03), C5 (3437-SIN-06), and C4-Fuyang RG virus. RD cells were infected with EV71 and the supernatants were collected and clarified. Blots were incubated with 7C7 followed by HRP-coupled secondary antibody. Bands for VP0 (35 kDa) and VP2 (28 kDa) could be detected in all strains except the RG virus carrying the serine to threonine mutation in the 7C7 epitope.

To corroborate the findings of the mutational analysis conducted above, we infected RD cells with EV71 viruses from different subgenogroups and performed a Western blot. The selected wild-type strains were A (BrCr), B2 (7423-MS-87), B4 (HFM41), B5 (NUH0083-SIN-08), C1 (Y90-3761), C4 (75-Yamagata-03), and C5 (3437-SIN-06). In addition, cells were infected with reverse genetically engineered (RG) virus of the C4-Fuyang-08 strain carrying the serine to threonine mutation in the 7C7 epitope. Cytopathic effect could be observed in more than 90% of the cells at 48 h post-infection when the supernatants were collected. Cell debris was removed by a clarification spin and microfiltration through a 0.2 um cut-off filter and virus was then inactivated with BEI. Inactivated virus was concentrated by ultraspin, and subjected to SDS-PAGE. Western blotting using 7C7 as primary antibody and HRP-labeled secondary antibody was performed and the signal was detected by ECL. In all the strains carrying the ED/NSHP epitope, two specific bands corresponding to VP0 and VP2 could be detected (Figure 3, arrows), while there was no signal for the C4 strain RG virus. IFA gave the same results (Additional file 1).

### Serine to threonine mutation at position 144 of VP2 abolishes antigenicity

**Figure 4:**
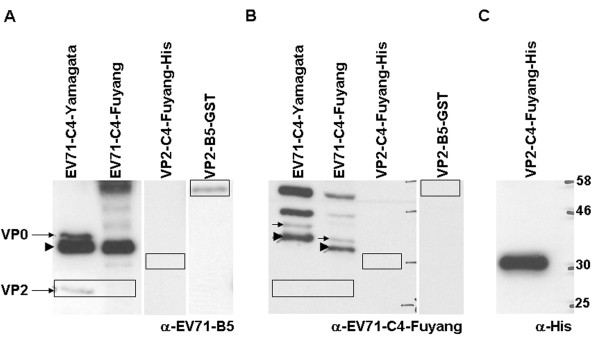
**Antigenicity of VP2 is abolished in S/T(144) mutant EV71 strains**. Polyclonal sera from mice injected with either wild-type EV71-B5 or EV71-C4-Fuyang RG virus were tested for the presence of anti-VP2 antibodies. Western blots were performed on samples of EV71-C4-Yamagata virus, EV71-C4-Fuyang virus, recombinant VP2-C4-Fuyang protein carrying a His tag, and recombinant VP2-B5-GST fusion protein protein. (A) Serum from EV71-B5 injected mice was used as a control. This serum could recognize VP1 in both C4 strains (arrowhead). It was also able to detect VP0 and VP2 in the EV71-C4-Yamagata stain carrying the consensus epitope EDSHP (arrows). There was no recognition of VP0 and VP2 in EV71-C4-Fuyang or of full length VP2-C4-Fuyang protein containing the S/T(144) mutation. In contrast, recombinant VP2-GST fusion protein from B5 strain was detected. (B) Serum from mice injected with EV71-C4-Fuyang was tested for the presence of anti-VP2 antibodies. Again, VP1 protein could be detected in both C4 strains (arrowheads). Additionally, there might be recognition of VP0 (arrows) but the serum contained no Abs against VP2 of either C4 virus strain, recombinant VP2-C4-Fuyang protein, or VP2-B5 protein (boxes). (C) Expression control for VP2-C4-Fuyang protein using anti-His antibody.

In order to investigate the role of the serine to threonine mutation in some recent EV71-C4 China strains, we compared polyclonal antisera of mice immunized with either EV71-B5 (used to isolate MAb 7C7) or RG virus EV71-C4-Fuyang, carrying the mutation. The antisera were assayed for their ability to detect VP2 protein in Western blots. As targets, we loaded recombinant full length VP2 protein with His tag cloned from EV71-C4-Fuyang strain, recombinant full length VP2-GST fusion protein cloned from EV71-B5 strain, wild-type virus EV71-C4-Yamagata which carries the consensus epitope EDSHP and RG virus EV71-C4-Fuyang with the EDTHP mutation. The polyclonal serum from EV71-B5 injected mice could recognize VP1 in both C4 strains (Figure 4A, arrowhead). It was also able to detect VP0 and VP2 in the C4-Yamagata strain (Figure 4A, arrows) and detected the recombinant VP2-B5 protein (Figure 4A, box 3). Anti-EV71-B5 serum however could not detect VP0 or VP2 in the in C4-Fuyang RG virus nor could it detect the full length VP2-Fuyang protein (Figure 4A, lanes 2 & 3). Next, we tested polyclonal serum from mice injected with C4-Fuyang RG virus. Again, VP1 was detected in both C4-Yamagata and C4-Fuyang (Figure 4B, arrowheads) and there was recognition of a putative VP0 band (Figure 4B, arrows). However, the C4-Fuyang serum contained no antibodies against VP2 (Figure 4B, boxes). The expression level of VP2-C4-Fuyang protein was monitored by anti-his antibody (Figure 4C). Taken together, these results show that there is only a single linear VP2 epitope on the EV71 virus and that the mutation of serine (144) to threonine (144) in this epitope confers a loss of VP2 antigenicity to some newly emerged C4-China strains.

### 7C7 is specific for EV71 and does not recognize coxsackieviruses

**Figure 5:**
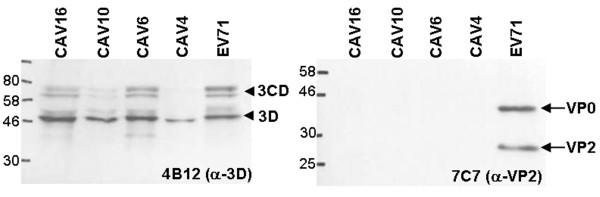
**7C7 does not detect coxsackieviruses**. RD cells were infected with either EV71-B5 strain, CAV16, CAV10, CAV6, or CAV4. Cells were incubated 48 h until CPE was observed. Supernatants were clarified and concentrated before blotting. As a control for CAV infection, blots were labeled with enterovirus A specific MAb 4B12 which detected its targets 3D (~52 kD, arrowhead) and 3CD (~73 kD, arrowhead) in all four tested coxsackievirus strains CAV16, 10, 6 and 4 as well as EV71. However, neither VP0 nor VP2 protein was detected by MAb 7C7 in these samples (arrows).

So far, no MAbs have been identified, that are specific exclusively for EV71 subgenogroups [35]. We therefore tested our new MAb 7C7 for cross reactivity to several strains of coxsackieviruses, namely CAV4, 6, 10 and 16 in both IFA (Additional file 2) and Western blot. The enterovirus A specific MAb 4B12, which reacts with the 3D polymerase and 3CD proteinase (in press), was used as a control to monitor the replication of the CAV. RD cells were infected with either EV71, CAV4, 6, 10 or 16. CPE was apparent 48 h post infection when cell supernatants were collected and concentrated as described above. MAb 7C7 did not cross-react to CAV4, 6, 10 and 16 in Western blotting of concentrated virus particles (Figure 5). At first glance this result conflicted with our epitope analysis, but comparison of the six amino acids surrounding the minimal epitope, showed four substitutions in CAV16 versus EV71-A. The flanking region in all EV71 strains including BrCr is TGT-epitope-YKQ whereas the flanking region of our CAV16 strain is DGN-epitope-YVT. CAV16 has a threonine to aspartic acid (nucleophilic to acidic) and a threonine to asparagine (nucleophilic to amide) substitution on the 5' end of the minimal epitope and a lysine to alanine or valine plus a glutamine to threonine substitution (positive charge to hydrophobic, amide to nucleophilic respectively) at the 3'end (Table 1). These alterations might prevent the binding of MAb 7C7 to the minimal epitope due to sterical hindrance in the context of the whole CAV16 virus.

### Antigen-capture ELISA

**Figure 6:**
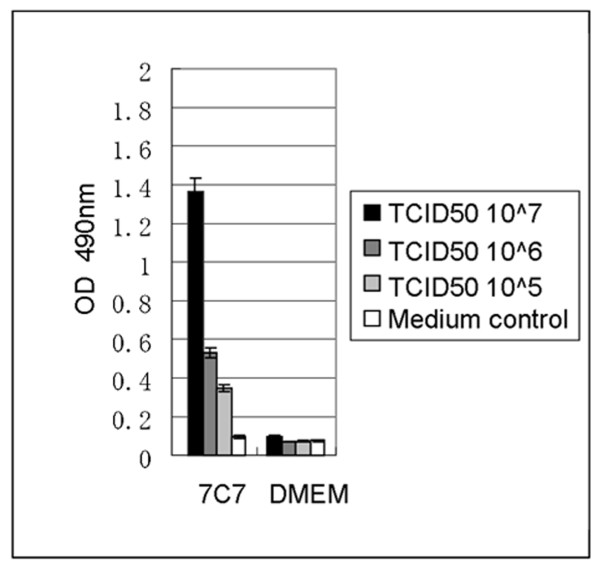
**Antigen capture ELISA**. 500 ng of rabbit polyclonal anti-EV71 antibody was coated in each well of the ELISA plate. EV71-B5 virus antigen was added at dilutions of 10^7, 10^6, and 10^5 TCID_50 _units per well and detected by 1 ug of MAb 7C7 followed by HRP labeled anti-mouse antibody. DMEM media instead of 7C7 was used as a negative control. O-phenylenediamine as a substrate was used for color development and the OD was measured at 490 nm. The anti-VP2 MAb 7C7 could detect as little as 10^5 TCID_50 _units of EV71 captured by polyclonal serum in this ELISA assay.

The MAb 7C7 was tested as a detection antibody in an AC-ELISA assay. First, we performed a checkerboard titration was performed. A signal to noise ratio of 3 was obtained for 100 ng-1ug capture serum and 1 ug of detection Ab. To evaluate the sensitivity of the AC ELISA, 500 ng of rabbit polyclonal anti-EV71 antibody (in house production) was coated in each well of the ELISA plate. Clarified supernatant from EV71-B5 infeceted RD cells was then added at dilutions of 10^7^, 10^6^, and 10^5 ^TCID_50 _units per well, and detected by MAb 7C7 followed by HRP-labeled anti-mouse antibody. DMEM media was used as negative control. Color was developed using freshly prepared substrate solution (o-phenylenediamine) and OD measured at 490 nm. The MAb 7C7, targeting the viral capsid protein VP2, was able to detect 10^5 ^TCID_50 _units of EV71 virus which corresponds to the amount detected by dot blotting. Again, the signal to noise ratio cut-off was set at 3 times (Figure 6).

## Discussion

A syndrome of rapid onset pulmonary edema and brainstem encephalitis has been observed in recent large outbreaks of HFMD in East Asia [15,18]. As these neurologic complications of HFMD are mainly caused by infection with EV71 [14,36], there is an urgent need to develop assays to diagnose EV71 early in infection and to distinguish HFMD caused by EV71 from the milder form caused by CAV16. A real time PCR based molecular test to detect virus from patients has been developed [37]. However, PCR tests are extremely sensitive and need extensive controls while antigen detection by MAbs has the advantage of the relative ease of sample handling and the use of less stringent procedures. Specific MAbs can then be used to develop a rapid test such as dip stick or lateral flow. In this study, we have characterized the epitope of the EV71-specific monoclonal antibody 7C7 in detail. The linear epitope of 7C7 was mapped to amino acids 142-146 within the VP2 capsid protein and further characterized by mutational analysis. In Western blots, 7C7 could detect bands for both VP2 and its precursor VP0. MAb 7C7 was also able to detect all EV71 subgenogroups by IFA and Western blot, with the exception of some recent C4 China strains that carry the S/T(144) mutation in VP2. The MAb did not cross-react to coxsackieviruses CAV16, 10, 6, and 4 and was used successfully as a capture antibody in an AC-ELISA.

### VP2 is an immunogen of EV71 and its epitope is located in the E-F loop

The major antigenic determinants of picornaviruses are usually found on the VP1 capsid protein. This poses the problem of generating universal antibodies against all subgenogroups of a virus strain, as the VP1 sequence also carries most of the mutations defining virus classification. VP0 and VP2 have been described to carry antigenic determinants in other picornaviruses and might be more useful for diagnostic purposes due to their higher sequence conservation between subgenogroups. VP0/VP2 and VP3 have been shown to form antigenic structures in a variety of picornavirus family members such as poliovirus 1, poliovirus 3, coxsackievirus A9 (CVA9), human rhinovirus 14, and foot-and-mouth disease (FMDV) virus [26,38-40]. These include conformational neutralizing epitopes spanning more than one capsid protein such as N-AgII and N-AgIV of the Sabin polio virus [27] or the D epitope of FMDV [41], neutralizing epitopes of VP2 in CVA9 [28], as well as linear epitopes of VP2 in human parechoviruses [30]. The latter has been used to create a serological assay to detect parechovirus antibodies in human sera [32] confirming the feasibility of exploiting VP2 antigenicity for virus detection.

In our study, we injected mice with inactivated EV71-B5 virus and isolated a MAb that recognized two protein bands of around 35 kDa and 28 kDa in a Western blot, corresponding to VP0 and VP2. This demonstrates the immunogenicity of the EV71 VP2 protein in the context of the virion. Sequential deletions of the VP2 capsid protein were used to exactly map the epitope of MAb 7C7 to amino acids 142 to 146 of VP2. This epitope lies in the E-F loop protruding out of the β-sheets encompassing amino acids 130 to 170, as has been described for the VP2 epitopes of other picornaviruses [26,28,38,42]. In addition, a recent paper described the epitope of the commercially available anti-VP2 MAb979 (Chemicon, USA) roughly as amino acids 136-150 which encompasses the exact epitope EDSHP that we identified. This approximated epitope has been found to be non-neutralizing when injected into mice, corresponding to our findings [43].

### Serine to threonine mutation at position 144 of VP2 abolishes antigenicity of the EV71 VP2 protein

Blast analysis of the exact VP2 epitope revealed two single amino acid variations within the EV71 family: D/N (143) was found in the EV71-A isolate BrCr while the S/T(144) substitution was limited to some EV71-C4 China strains. Mutational analysis of the epitope showed that the D/N (143) mutation of the EV71 subgenogroup A did not interfere with antibody recognition, while the S/T(144) mutation abolished antibody binding. By analyzing polyclonal serum from mice immunized with EV71-C4 RG virus carrying the S/T(144) mutation, we proved that no antibodies were formed against VP2 in these mice. Our results clearly show that there is only a single linear VP2 epitope on the EV71 virus and that the mutation of S/T(144) confers a loss of VP2 antigenicity to recently emerged EV71-C4 strains from China. A total of 30 published sequences were found to carry this mutation, while the majority of C4 strains from China and other countries had the conserved epitope and could be recognized in our assays.

### MAb 7C7 does not cross-react with CAV16 and can be used in an AC-ELISA

Many mABs generated against enteroviruses turned out to cross-react to one or more different species and so far, no mABs have been described that can distinguish EV71 from CAV16 virus. In the only EV71 study where cross-reactivity was tested, the MAb raised against the EV71 VP1 protein did indeed cross-react [44]. A blast of the 7C7 epitope on the NCBI database revealed its high specificity: Besides EV71, only CAV16 and the human echovirus 30 sequences carried related epitopes. Mutational analysis of the 7C7 minimal epitope showed recognition of the major EV71 epitope EDSHP as well as ENSHPP of EV71-A (BrCr) and CAV16. IFA and Western blotting of wild-type virus strains, however, showed that 7C7 exclusively recognized EV71 and did not cross-react to CAV16. Sequence analysis of the amino acids immediately surrounding the minimal epitope ENSHPP of CAV16 revealed four substitutions, including a nucleophilic to acidic and a positive charge to hydrophobic change, which might cause sterical hindrance to the binding of the MAb to the whole CAV16 virus. To test the suitability of MAb 7C7 in antigen detection, we performed an AC-ELISA using polyclonal anti-EV71 serum from rabbit as a capture Ab for EV71 virus particles and 7C7 as a detection Ab. We successfully detected a minimum of 10^5 ^TCID_50 _units of EV71 virus corresponding to the detection limit in the dot blot assay. Even tough 7C7 is not the perfect candidate for universal diagnosis, it nevertheless is the only MAb described so far that can differentiate between CAV16 and EV71.

## Conclusions

Detailed mapping of the epitope of MAb 7C7 allowed us to conduct mutational analysis with regard to EV71 and CAV lineages. We were able to demonstrate that there is only a single linear epitope on the VP2 protein of EV71, namely ED/NSHP comprising amino acids 142-146. The S/T(144) mutation in this epitope confers a loss of antigenicity to the VP2 protein of some newly emerged EV71-C4 strains from China. The MAb 7C7 was shown to exclusively recognized EV71 as it did not cross-react to coxsackieviruses 4, 6, 10, and 16 in IFA and Western blots. 7C7 is thus the first MAb that can differentiate CAV16 from EV71 and it was used successfully in an AC-ELISA.

## Materials and methods

### Viruses and cells

Wild-type enterovirus 71 and coxsackievirus strains were received from the Human Genome Laboratory, Department of Microbiology, Yong Loo Lin School of Medicine, National University of Singapore, Singapore. Additionally, the RG virus EV71-C4-Fuyang (NCBI accession #EU703813.1) has been constructed in the lab by reverse genetics. All virus strains have been propagated in rhabdomyosarcoma (RD) cells grown in DMEM medium (Gibco) with 10% fetal bovine serum (FBS). Virus was added to the culture medium, incubated at 37°C for 48 h when over 90% of cytopathic effect (CPE) was observed. The supernatant was collected and the virus activity was tested on RD cells in an end-point dilution assay (Reed & Muench, 1938) to calculate the tissue culture infective dose (TCID_50_). Before further experimentation, virus was inactivated with BEI. A 0.2 M solution of 2-bromoethylamine hydrobromide in 0.4 M NaOH (BEI) was prepared and incubated overnight at room temperature. BEI (3.2 mM) was then added to the cell supernatant containing virus and incubated for 48 h at 37°C. BEI was neutralized with 1/10 total volume of 1 M sodium thiosulfate. Cell supernatant was then clarified by centrifugation at 7,500 g for 30 min and filtration through a 0.2 um cut-off filter (Millipore). The virus was concentrated 20 times by an ultraspin at 100,000 g for 3 h and resuspended in PBS.

### Preparation and purification of MAb 7C7 from whole EV71 virus

All animal experiments were carried out in accordance with the Guides for Animal Experiments of the National Institute of Infectious Diseases (NIID), and experimental protocols were approved by the Institutional Animal Care and Use Committee of the Temasek Life Sciences Laboratory. Hybridomas secreting specific MAbs were derived from BALB/c mice which had been immunized twice subcutaneously with inactivated and concentrated EV71-B5 strain in 0.1 ml of PBS, emulsified with an equal volume of adjuvant (SEPPIC, France). An intraperitoneal booster of the same dose of virus was administered 3 days before splenocytes were fused to the SP2/0 myloma cells, as previously described [45]. Hybridomas were screened by IFA of Vero cells infected with EV71-B5. Clones identified to produce specific antibody were then sub-cloned by limiting dilution, and expanded in 75 cm^2 ^flasks. One week later, the hybridoma suspension was harvested and cell debris pelleted by centrifugation at 400 g for 10 min, followed by collection of the supernatant and storage at -20°C. Ascites fluid was produced by intraperintoneal injection of hybridoma cells in balb/c mice followed by MAb purification with the Montage Prosep-A antibody purification kit (Millipore, USA). MAb was isotyped using Isostrip (Santa Cruz, USA) and concentrations were determined spectro-photometrically (Nanodrop, DE).

### Immunofluoresence Assay (IFA)

For antibody screening, Vero African green monkey kidney cells were seeded overnight onto 96-well plates in DMEM with 10% FBS at approximately 90% confluence and infected with 100 μl virus at 10^5 ^TCID_50_. Vero cells were incubated at 37°C for 48 h when CPE could be observed, and for 15 min in 4% paraformaldehyde/PBS (pH 7.4) and permeabilized with 0.1% Triton-X/PBS for 15 min. After blocking with 10% fetal bovine serum/PBS for 1 h, cells were incubated in hybridoma cell supernatant for 1 h followed by three 15 min washes in 0.1% Tween/PBS. FITC-coupled secondary antibody was applied for 1 h, again followed by three 15 min washes in 10% Tween/PBS. All steps were performed at room temperature. Results were documented in an inverted microscope (Olympus) with Nikon ACT-1 software.

### Western Blotting

Protein samples were obtained from concentrating EV71 virus particles by ultraspin and from E. coli cell lysis and sonication (GST-fusion proteins, His-tagged proteins). Protein samples were denatured in loading buffer (Tris pH 6.8, Glycerol, 20% SDS) with 5% β-mercaptoethanol, and heated at 95°C for 5 min. 10-12% SDS-PAGE gels were prepared according to standard protocols, and electrophoresed at 100 volts for 3 h. Proteins were then transferred to nitrocellulose membranes (TransBlot, BioRad) by wet western blotting at 100 volts for 2 h in transfer buffer (12.5 mM Tris, 96 mM Glycine, 10% Methanol). Blots were blocked in 5% blotting grade milk/PBS and incubated in a 1:100 dilution of polyclonal anti-EV71 serum (Figure 1B), or hybridoma cell supernatant for 1 h each. Membranes were washed three times for 15 min each in 0.1% Tween-20/PBS before incubation in horse-radish-peroxidase coupled secondary antibody diluted 1:10,000 (Dako Cytomation) for 1 h. Membranes were washed again three times for 15 min each in 0.1% Tween-20/PBS followed by incubation with chemoluminescent ECL reagent (Amersham) and detection with Hyperfilms (Amersham). All steps were performed at room temperature.

### Dot Blotting

Wild-type virus strain C4-Yamagata propagated in RD cell supernatants was serially diluted to a TCID_50 _ranging from 10^6.6 ^to 10^3.6^. 50 ulof virus dilution was mixed with 200 ulof transfer buffer (20 mM Tris pH 7.5, 500 mM NaCl), and applied to a dot blot apparatus (BioRad) containing a nitrocellulose membrane (TransBlot, BioRad). A vaccum pump was used to adsorb the sample to the membrane, and an additional 100 ulof transfer buffer was added as a wash. The blot was then processed as a Western blot as described earlier. Signals were developed by ECL (Amersham). All steps were performed at room temperature.

### Construction of GST fusion proteins with fragments of VP2 sequence for epitope mapping

Total RNA was extracted from EV71-B5 with RNAeasy kit (Qiagen, Germany) according to manufacturer's protocol. Full length VP2 gene was then amplified by reverse transcription PCR and cloned into pGEX-4 T-1. Fragments of 3' end-truncated VP2 gene were then amplified with the respective primers using full length pGEX-4 T-1-VP2 as template. Additionally, alternative epitopes were expressed by incorporating the specific DNA sequence in the sense primer and using pGEX-4 T-1 as the template. PCR products were double-digested with EcoRI and XhoI and ligated into pGEX-4 T-1. Recombinant vectors were sequenced and transformed into *E. coli *BL-21 cells for the expression of glutathione S-transferase (GST) fusion proteins. The positive transformants were grown in Luria-Bertani (LB) medium with 100 μg/ml of amplicillin at 37°C on a shaker until an OD_600 _of 0.6- 0.8 was reached. Cells were then induced for 3 h by 1 mM isopropyl-b-D-thiogalactopyranoside (IPTG) at 37°C and sonicated for 5 min. These GST fusion proteins were detected with anti-GST MAb by Western blot hybridization. Alternatively, the VP2-C4-Fuyang protein was amplified from a synthetic gene (GenScript, Nanjing, China) designed according to the published sequence of the EV71-C4 Fuyang-08 subgenogroup (NCBI accession # EU703813.1). It was cloned into the pet-28a vector (Novagen, USA) with an N-terminal His-tag. After transformation and sequencing of positive clones, the protein was expressed in *E. coli *BL-21 cells as described above.

### Antigen capture ELISA

96-well, round-bottom microtiter plates (Nunc, Roskilde, Denmark) were coated with 500 ng per well of polyclonal rabbit anti-EV71-B5 capture antibody (raised in house) in 100 ul of carbonate buffer (73 mM sodium bicarbonate and 30 mM sodium carbonate, pH 9.7) overnight at 4°C. After each incubation with antibody or antigen, the plates were washed twice with PBST, followed by two washes with PBS. The antibody-coated plates were blocked with 100 ul of blocking buffer (PBS containing 5% milk) for 1 h at room temperature, and then incubated at 37°C for 1 h with 100 ul of virus-containing clarified supernatant diluted in PBST. Virus binding was detected by incubation with 1 ug of purified monoclonal detection antibody for 1 h at 37°C. 100 ul of 1:3000 HRP conjugated anti-mouse antibody (Dako, Denmark) was used for labeling for 1 h at 37°C, and 100 ul freshly prepared substrate solution (o-phenylenediamine-dihydrochloride; Sigma, USA) was added as chromogen. The reaction was stopped by 0.1 M sulfuric acid, and the optical density at 490 nm respectively was measured with an ELISA reader (Tecan, Switzerland). The detection limit was determined as the optical density value that yielded a signal-to-noise ratio of 3.

## Competing interests

The authors declare that they have no competing interests.

## Authors' contributions

TK participated in the design and coordination of the study, performed AC-ELISA optimization and prepared the manuscript. QJ isolated the MAb 7C7 and conducted IFA and Western blot experiments for its characterization. He was involved in epitope mapping and sequence alignments. XFL conducted the epitope mapping, dot blotting, Western blotting and IFA of 7C7. Both QJ and XFL prepared virus stocks for IFA and blotting experiments. FH conducted the sensitivity assay for the 7C7 AC-ELISA and TM generated the C4-Fuyang EV71 virus by reverse genetics. VC supplied virus strains. JK oversaw all the experiments conducted and helped to draft the manuscript. All authors read and approved the final manuscript.

## Supplementary Material

Additional file 1 **Specificity of 7C7 to EV71 subgenogroups by IFA**. To corroborate the findings of the Western blot, we infected African green monkey kidney cells (Vero) with EV71 viruses from different subgenogroups and performed IFA. The selected wild-type strains were A (BrCr), B2 (7423-MS-87), B4 (HFM41), B5 (Malaysia, unpublished sequence), B5 (NUH0083-SIN-08), C1 (Y90-3761), C2 (NUH0075-SIN-08), C4 (75-Yamagata-03), and C5 (3437-SIN-06) previously grown in rhabdomysarcoma (RD) cells. Furthermore, Vero cells were infected with reverse genetically engineered (RG) virus of the C4-Fuyang-08 strain carrying the serine to threonine mutation in the 7C7 epitope. Cytopathic effect could be observed at 48 h post-infection when cells were fixed and labeled with 7C7 followed by anti-mouse FITC labeled secondary antibody. All tested wild-type strains were positively identified by our MAb whereas the RG virus could not be detected. Click here for file

Additional file 2 **7C7 does not recognize coxsackieviruses by IFA**. IFA with MAbs 7C7 (A) and 4B12 (B). Vero cells were infected with either EV71-B5 strain, CAV16, CAV10, CAV6, or CAV4. Cells were incubated 24 h until CPE was observed (see bright field images) at which time point the cells were fixed and processed for IFA. (A) IFA was conducted with MAb 7C7 and FITC labeled secondary antibody. No labeling was observed in the CAV infected cells. (B) As a control for CAV infection, cells were labeled with enterovirus A specific MAb 4B12 (in house production), followed by anti-mouse FITC secondary antibody. All CAV strains were detected, confirming virus replication. Click here for file
