# Supplementary figures and images for: Characterization and specificity of the linear epitope of the enterovirus 71 VP2 protein
Source: Virol J. 2012 Feb 24;9:55. doi: 10.1186/1743-422X-9-55 (PMC3307493; doi:10.1186/1743-422X-9-55)

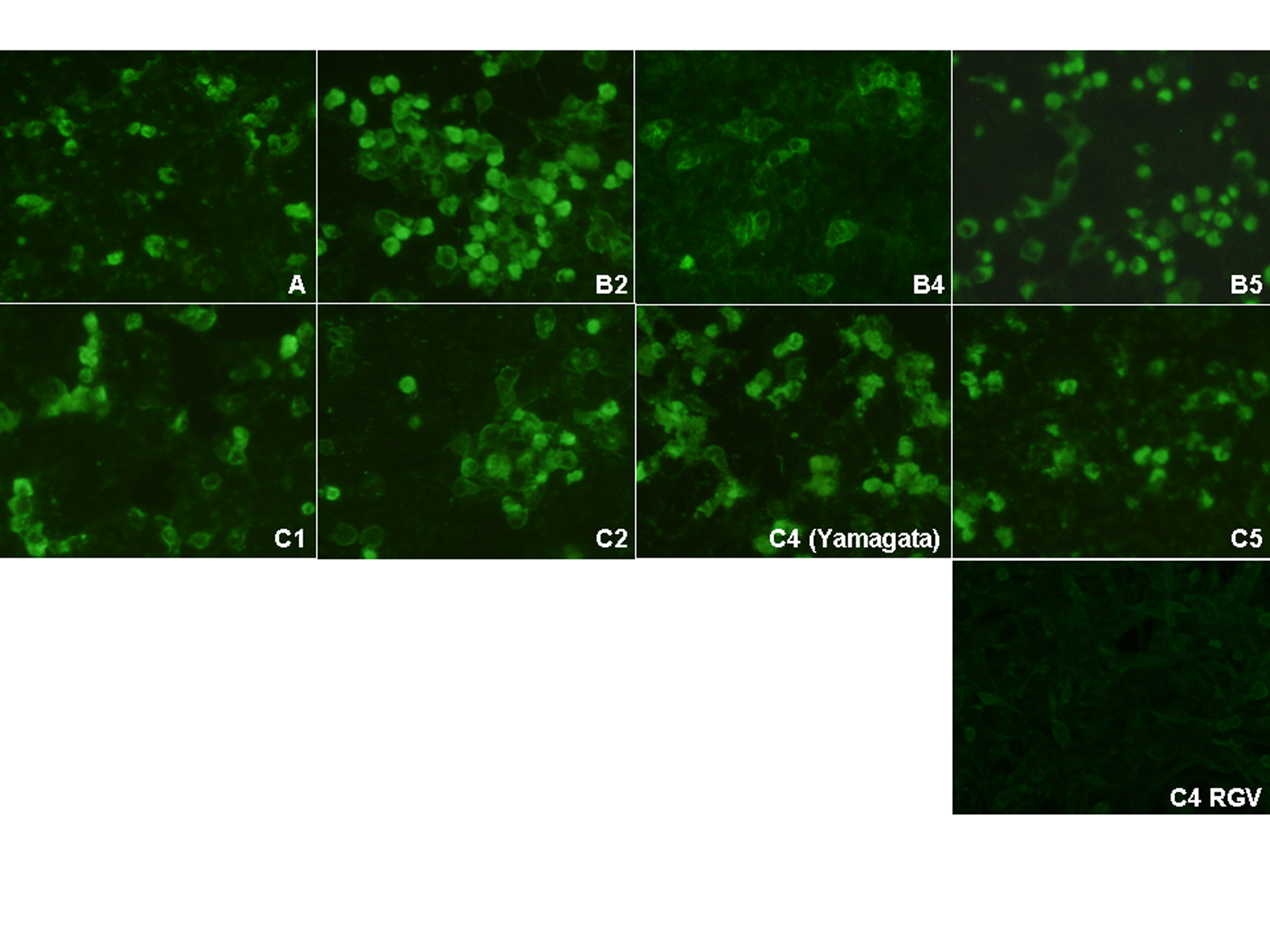

Supplement: Additional file 1 — Specificity of 7C7 to EV71 subgenogroups by IFA. To corroborate the findings of the Western blot, we infected African green monkey kidney cells (Vero) with EV71 viruses from different subgenogroups and performed IFA. The selected wild-type strains were A (BrCr), B2 (7423-MS-87), B4 (HFM41), B5 (Malaysia, unpublished sequence), B5 (NUH0083-SIN-08), C1 (Y90-3761), C2 (NUH0075-SIN-08), C4 (75-Yamagata-03), and C5 (3437-SIN-06) previously grown in rhabdomysarcoma (RD) cells. Furthermore, Vero cells were infected with reverse genetically engineered (RG) virus of the C4-Fuyang-08 strain carrying the serine to threonine mutation in the 7C7 epitope. Cytopathic effect could be observed at 48 h post-infection when cells were fixed and labeled with 7C7 followed by anti-mouse FITC labeled secondary antibody. All tested wild-type strains were positively identified by our MAb whereas the RG virus could not be detected. [file 1743-422X-9-55-S1.TIFF]

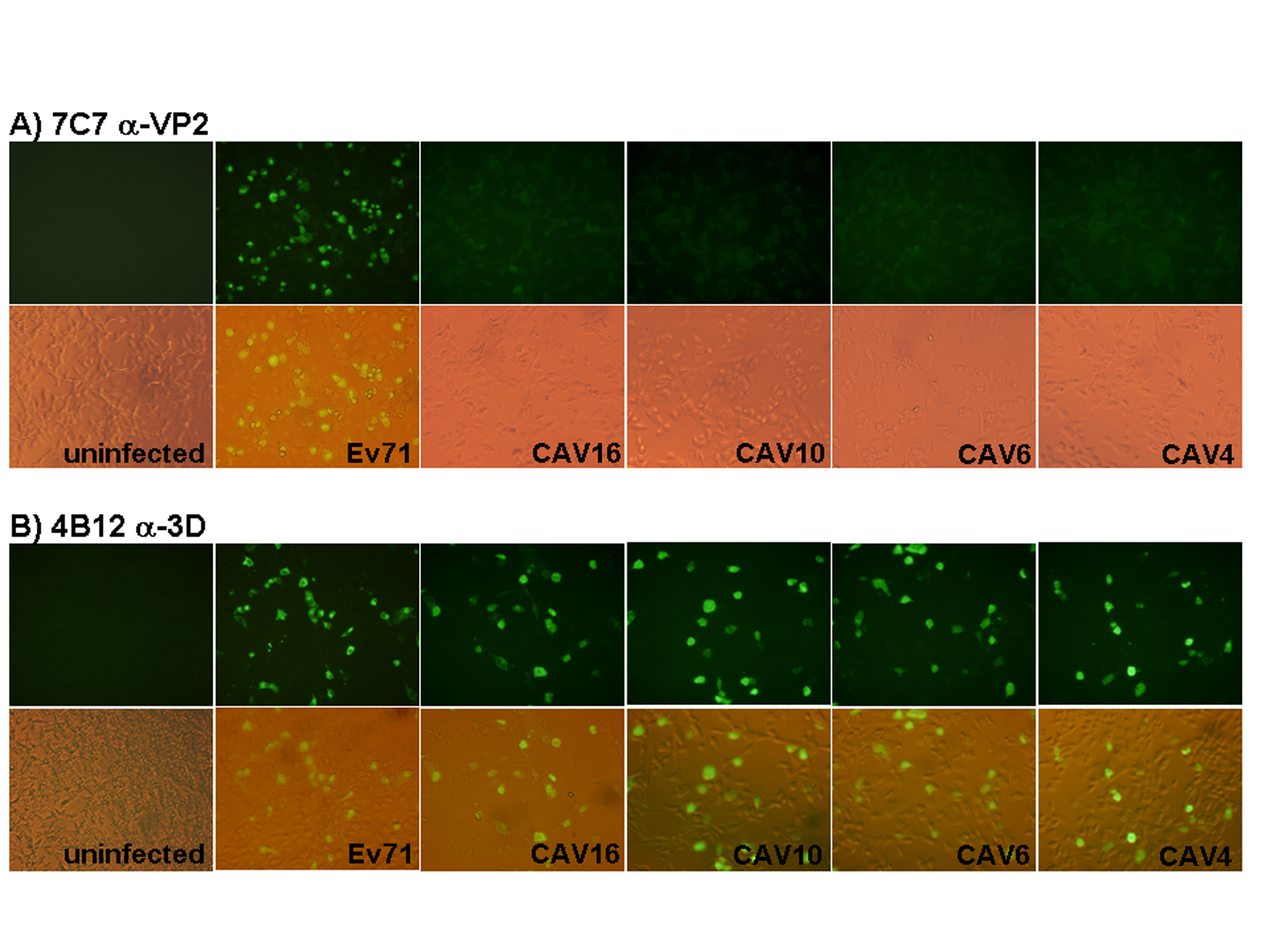

Supplement: Additional file 2 — 7C7 does not recognize coxsackieviruses by IFA. IFA with MAbs 7C7 (A) and 4B12 (B). Vero cells were infected with either EV71-B5 strain, CAV16, CAV10, CAV6, or CAV4. Cells were incubated 24 h until CPE was observed (see bright field images) at which time point the cells were fixed and processed for IFA. (A) IFA was conducted with MAb 7C7 and FITC labeled secondary antibody. No labeling was observed in the CAV infected cells. (B) As a control for CAV infection, cells were labeled with enterovirus A specific MAb 4B12 (in house production), followed by anti-mouse FITC secondary antibody. All CAV strains were detected, confirming virus replication. [file 1743-422X-9-55-S2.TIFF]
